# Metastasis-associated upregulation of ER-Golgi trafficking kinetics: regulation of cancer progression via the Golgi apparatus

**DOI:** 10.18632/oncoscience.426

**Published:** 2018-06-25

**Authors:** Breege V. Howley, Philip H. Howe

**Affiliations:** Dept. of Biochemistry and Molecular Biology, Medical University of South Carolina, Charleston, SC 29425, USA

**Keywords:** metastasis, ER-Golgi trafficking, stress response

The Golgi apparatus acts as a signaling hub, integrating stimuli from multiple sources to control protein and lipid post-translational modification and transport, impacting membrane composition, secretion and receptor cycling. Within the linked cisternae that comprise the Golgi ribbon, post-translational modifications, including N-glycan modification, O-linked glycosylation and sulfation, occur before transport of cargo to the Trans-Golgi Network (TGN) for sorting. Owing to the Golgi's ability to process and traffic a large portion of the proteome and regulate multiple processes in the cell including mitosis, apoptosis and migration, it is not surprising that dysregulation of Golgi function is linked to several pathologies including Parkinson's and Alzheimer's disease and cancer.

**Figure 1:**
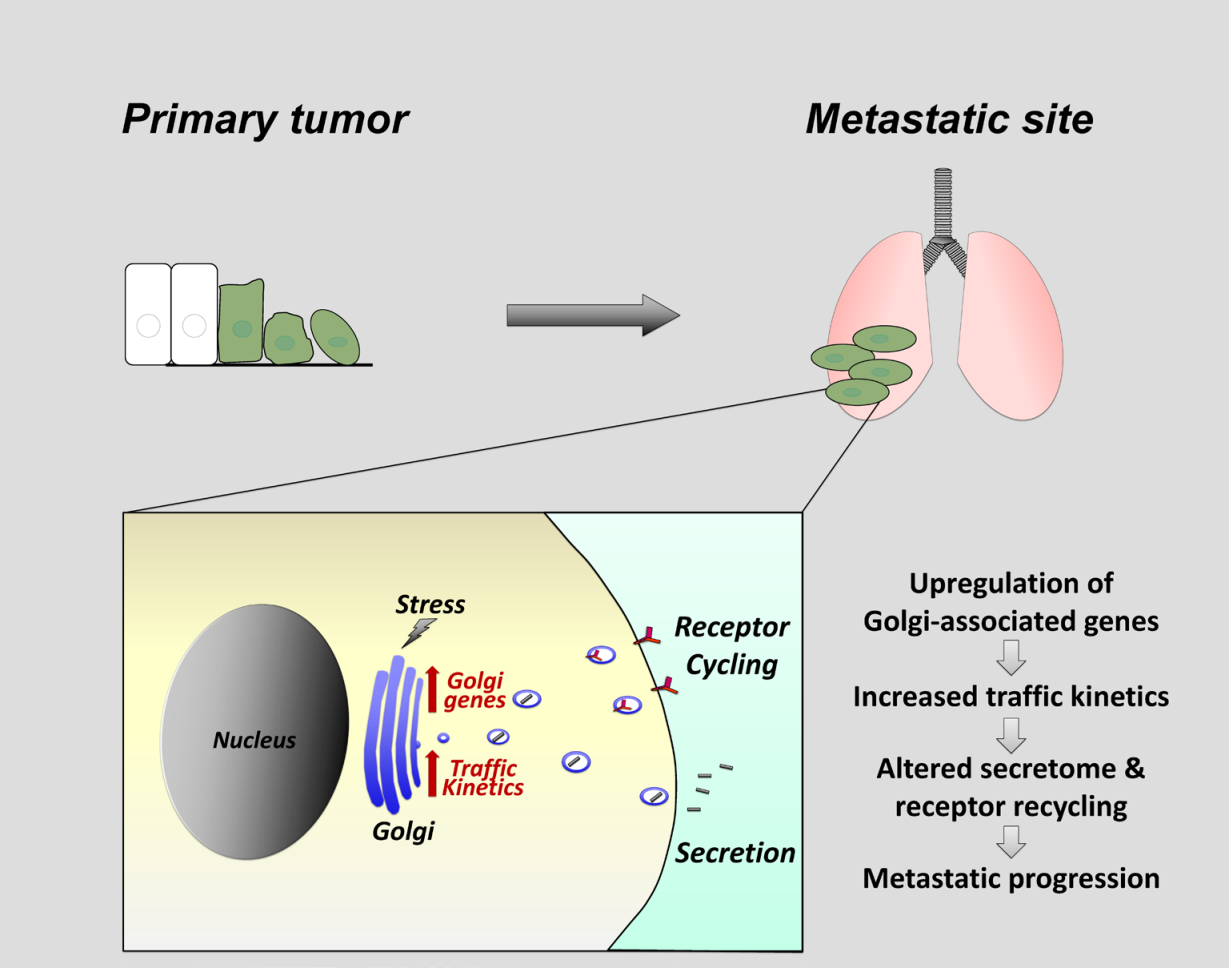
Model of ER-Golgi trafficking gene up-regulation in metastatic cells CREB3 mediated up-regulation of Golgi-associated genes promotes increased ER-Golgi trafficking and altered secretion and receptor cycling. This in turn increases cell-matrix adhesion, invasion and migration aiding metastatic progression. We propose that activation of CREB3 transcription factors in metastatic cells may occur in response to Golgi stress.

Recent studies have linked alterations in Golgi function to the metastatic progression of cancer cells. For example, GOLPH3, an oncogenic Golgi-resident protein that is a key regulator of Golgi morphology and function, is upregulated in several cancer types and this increase in expression correlates with poor prognosis [1]. PITPNC1, in complex with RAB1B, has been shown to promote Golgi extension and secretion in malignant cells by increasing levels of GOLPH3 [2]. In a separate study, Golgi compaction, driven by the scaffolding protein PAQR11, has been described in cells that have undergone an Epithelial to Mesenchymal transition (EMT), a process linked to metastatic progression [3]. Our recent findings reveal a CREB3-regulated ER-Golgi trafficking gene signature that includes ARF4, COPB1 and USO1 in mammary epithelial cells isolated from lung metastases [4]. Silencing of genes in this signature altered transport kinetics and secretion in metastatic cells; which in turn modulated cell-matrix adhesion, migration and invasion *in vitro* and lung metastasis *in vivo* (Figure 1).

How the Golgi apparatus integrates signals to alter morphology and trafficking kinetics under physiological and pathological conditions is poorly defined. A Golgi stress response has been proposed which may be activated by stimuli including amino acid depletion, pathogen infection and lipid content at Golgi vesicles; we propose that a similar stress response is activated in metastatic cells leading to an up-regulation of Golgi-associated genes in order to increase Golgi function. The downstream effectors of the Golgi stress response remain elusive; ELK1, GABPA/B, and ETS1 activation by MAPK signaling and increased ATF4 expression via PERK are reported to regulate the response induced by Golgi stressors, such as monensin [5, 6]. In addition, TFE3 and MLX bind a Golgi apparatus stress response element (GASE) in the promoter of Golgi-associated genes. We have observed increased activation of ER-localized CREB3 and CREB3L2 in metastatic cells, which control expression of the Golgi-associated genes ARF4, COPB1 and USO1 [4]. These findings are consistent with previous research demonstrating CREB3 regulation of ARF4 in response to Golgi stress [7]. Interestingly, activation of CREB3 and CREB3-like transcription factors require proteolytic processing which occurs at the Golgi prior to translocation to the nucleus. However, the stimuli and escort proteins needed to activate these factors in response to Golgi stress requires further characterization.

A better understanding of how Golgi function is altered during cancer progression may lead to novel prognostic tools and therapeutic interventions. Expression of Golgi-associated genes, such as GOLPH3, GP73 and PAQR3, is reported to be associated with patient survival, and therefore may be used as prognostic markers in cancer. In addition, altered Golgi function during metastatic progression may be harnessed therapeutically. We have observed sensitization to retrograde ER-Golgi transport inhibition by brefeldin A (BFA) in metastatic cells, which appears to be partially due to an upregulation of retrograde trafficking genes, such as ARF4 [4]. Thus, the development of more selective and bioavailable modulators of ER-Golgi trafficking may be used to target malignant cells that have up-regulated this pathway. Further studies focused on elucidating the regulatory mechanisms controlling Golgi function are warranted and may lead to therapeutic targeting of discrete components of this complex pathway.
